# A Case Report of Flecainide Toxicity With Review of Literature

**DOI:** 10.7759/cureus.22261

**Published:** 2022-02-15

**Authors:** Pratik Khatiwada, Lindsey Clark, Arjun Khunger, Bhimesh B Rijal, Jody Ritter

**Affiliations:** 1 Department of Internal Medicine, Memorial Healthcare System, Hollywood, USA; 2 Primary Care, Rocky Mountain Primary Care, Lakewood, USA; 3 Department of Cardiology, Memorial Healthcare System, Hollywood, USA

**Keywords:** drug overdose, intravenous fat emulsion, anti-arrhythmic drugs, cardiovascular toxicity, flecainide

## Abstract

Flecainide is an anti-arrhythmic drug with a narrow therapeutic index. Flecainide toxicity is rare, but the mortality is high. This case demonstrates the use of intravenous fat emulsion therapy in conjunction with intravenous sodium bicarbonate treatment for flecainide toxicity.

A 50-year-old male with atrial fibrillation and taking flecainide 75 mg twice daily presented to Emergency Department after ingesting 1125 mg of flecainide, in a suicide attempt. An electrocardiogram (ECG) on arrival showed bradycardia, wide QRS complex, prolonged QTc interval. Treatment for flecainide poisoning with intravenous sodium bicarbonate was initiated. On day two, the patient had a cardiac arrest secondary to ventricular tachycardia. After successful defibrillation, the patient had persistent bradycardia and hypotension. Administration of a 20% lipid emulsion bolus, followed by continuous infusion for three hours, resulted in conversion to normal sinus rhythm.

This case illustrates the successful treatment of flecainide toxicity with intravenous fat emulsion therapy. To our knowledge, this is the second case that used fat emulsion without concomitant extracorporeal life support. Due to its low prevalence and the fact the lipid emulsion is often used in conjunction with other treatments, there are no randomized clinical trials on the isolated efficacy of lipid infusion. The best treatment is unknown. Given its high mortality, early detection and treatment are paramount.

## Introduction

Flecainide is a Class IC antiarrhythmic drug and is an effective agent against supraventricular tachycardia and atrial fibrillation [1]. It causes rate-dependent slowing of phase 0 of the fast sodium channels, slowing the upstroke of the cardiac action potential, which slows the conduction of electrical impulses within the heart mostly on the His-Purkinje System and ventricular myocardium. Due to its narrow therapeutic index (0.2-1.0 mcg/mL), small differences in dose or blood concentration may lead to serious therapeutic failures and/or adverse drug reactions that are life threatening [2].

Flecainide overdose leads to excess cardiac sodium channel blockade causing delayed conduction, negative inotropy, and cardiac arrhythmias including atrioventricular (AV) nodal block, ventricular tachycardia/fibrillation, bradyarrhythmia, and asystole [2]. It manifests as prolonged PR interval, QRS, and QTc­ intervals on electrocardiogram (ECG). The diagnosis of flecainide toxicity is based on clinical suspicion and ECG findings, as the serum drug level may take days to weeks to show results.

The mainstay of treatment for flecainide overdose is sodium bicarbonate and supportive care [3]. Despite its narrow therapeutic index, flecainide toxicity is rare and treatment for its toxicity has not been formally studied. We present a case of intentional flecainide overdose treated with sodium bicarbonate, supportive care, and intravenous lipid emulsion. The use of intravenous lipid emulsion has been described for the treatment of cardio-toxic effects of local anesthetics or other lipophilic drugs like beta-blockers and calcium channel blockers. As flecainide is also a lipophilic drug, intravenous lipid emulsion was tried in our patient with prompt response to the treatment. A thorough review of the literature was conducted, and it revealed that our case is the seventh reported case to use lipid emulsion in conjunction with sodium bicarbonate administration for the treatment of flecainide toxicity.

## Case presentation

A 50-year-old Hispanic male with a medical history significant for hypertension, atrial fibrillation-flutter status post ablation twice on flecainide 75 mg two times a day and Eliquis 5 mg two times a day, and major depression and anxiety disorder on psychiatric medications presented to our Emergency Department (ED) with an acute change in his mental status onset two to three hours prior to arrival. Per emergency medical services (EMS), patient became very anxious, agitated, with increased tremors. Family members called EMS as they initially thought that the patient was having anxiety attack. His initial vital signs were pulse of 58 beats/min, blood pressure of 90/60 mm of Hg, respiratory rate 17 breaths/min, temperature 36.4 Celsius, and oxygen saturation of 99% on room air. In the ED, patient had an episode of witnessed generalized tonic-clonic seizure followed by unresponsiveness. The initial history and review of systems was limited because of the patient’s non-verbal status secondary to post-ictal stage. Seizure was treated with intravenous Ativan 2 mg once. On examination, patient was awake, not in any respiratory discomfort, drowsy with involuntary muscle twitching throughout his body. Lungs were clear to auscultation bilaterally, normal S1, S2 heart sounds, no added murmurs, rubs, or gallops. Cardiac monitor showed atrial flutter with bradycardia. Once his mental status improved in ED, he admitted to ingesting 15 pills of flecainide, 75 mg each (1,125 mg total), in a suicide attempt. He denied taking any additional medications apart from flecainide. 

**Figure 1:**
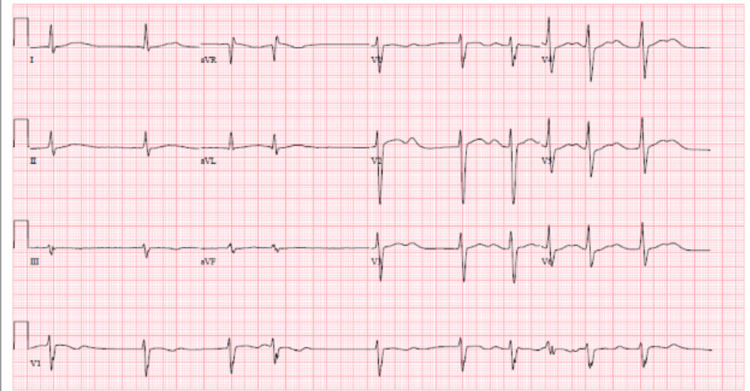
ECG on the second day of admission showing sinus bradycardia with AV dissociation, occasional premature ventricular contractions, QTc interval 544 milliseconds AV: atrioventricular

**Figure 2:**
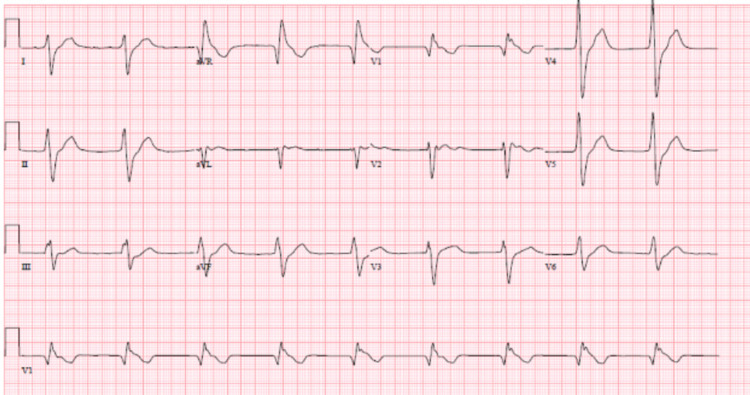
ECG upon arrival to ED, showing bradycardia, wide QRS complex, right bundle branch block, and prolonged QTc interval.

Initial laboratory studies were significant for hypokalemia with potassium of 3.2 mmol/L, elevated blood glucose (201 mg/dL), and elevated lactic acid level (2.9 mmol/L). Rest of the initial screening labs including complete blood count, comprehensive metabolic panel, magnesium, and ionized calcium levels were within normal limits. Troponin level was <0.0015 ng/ml. Blood ethanol was not detected; acetaminophen and salicylate level were not elevated. Urine drug screen was negative. CT brain without contrast ruled out any acute intracranial pathology. ECG on arrival to ED showed bradycardia, heart rate of 55 beats/min with wide QRS complex, QRS duration 200 ms and right bundle branch block (RBBB), prolonged QTc interval of 529 milliseconds (Figure 1). In the ED, intravenous (IV) fluid normal saline bolus was administered, IV sodium bicarbonate 150 mEq bolus and 18.75 mEq/hour infusion for the treatment of flecainide poisoning was given. This initiation of sodium bicarbonate is the mainstay of treatment in flecainide toxicity. Cardiology, Critical care, and Poison control consult were obtained because of bradycardia and shock from flecainide overdose. Patient’s blood pressure improved with IV fluids and bicarbonate drip and did not require vasopressors. His repeat blood pressure after IV fluids was 130/80 mm Hg. Patient remained bradycardic overnight with the heart rates in the 40s and telemetry showing idioventricular rhythm. Serial ECG monitoring for QTc interval was done (Figure 2).

On the second day of admission, patient had cardiac arrest from ventricular tachycardia requiring advanced cardiac life support (ACLS) and emergent defibrillation once. Post resuscitation, the patient was not comatose, and he did not require intubation. Further, patient converted back to sinus rhythm with bradycardia (heart rates were between 20 and 30 beats per minute). Due to the rate-dependent properties of flecainide, it was decided to leave the patient bradycardic given he had stable hemodynamics. IV magnesium infusion of 2 gram was given and patient was monitored closely for torsades.

**Figure 3:**
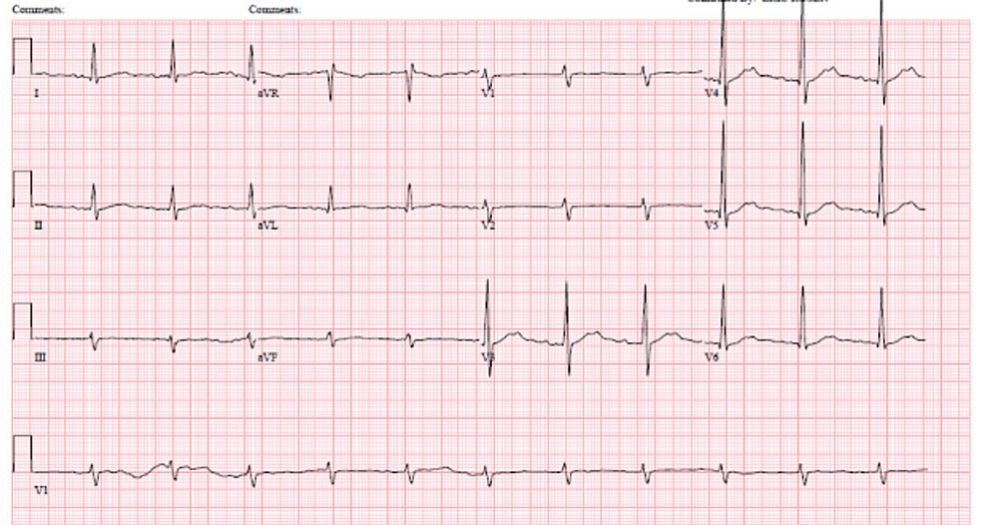
ECG on day four of admission showing sinus rhythm with first degree AV block, ventricular rate 68 bpm with PR interval 214 milliseconds, and QTc interval 518 milliseconds AV: atrioventricular

Treatment with IV fat emulsion was initiated on day two of admission as recommended by poison control due to the persistence of bradycardia and prolonged QT interval. Patient was administered with 20% lipid emulsion IV bolus at 1.5 mL/kg followed by IV infusion at 0.25 mL/kg/min. The patient weighed 68 kilograms and received total 311 ml of IV lipid (103 ml bolus over one hour then 288 ml over three hours). Alkalization with bicarbonate drip and other supportive treatment including correction of underlying electrolyte abnormalities and oxygen supplementation to avoid hypoxia were also continued simultaneously. A follow-up ECG showed sinus rhythm with 1st degree AV block and improvement in the QTc interval (Figure 3). After three days of intensive care unit management, the patient was downgraded to the intermediate care unit while being on bicarbonate drip as his QTc interval was still prolonged. Bicarbonate drip was later discontinued after the normalization of the QTc interval on fifth day of admission. Psychiatry consultation was obtained for his suicidal attempt, major depression, and anxiety disorder. Patient was later discharged to a psychiatry facility with recommendations to follow up with cardiology as an outpatient.

## Discussion

Flecainide is a class IC antiarrhythmic drug used in the treatment of supraventricular tachycardia and atrial fibrillation. Flecainide has a narrow therapeutic index (0.2-1.0 mcg/mL), which increases the potential for toxicity. Some patients may even experience symptoms of toxicity at lower serum levels (as low as 0.7 mcg/mL or higher) [4]. Although the reported prevalence of toxicity due to class IC antiarrhythmic is low (about 0.1% of all intoxications) [5], the reported mortality has been reported to be as high as 22% [6,7], which makes early detection and treatment paramount.

The adverse effects of flecainide are due to excess sodium channel blockade causing delayed conduction through the AV node, His-Purkinje system, and ventricles, which can present as AV block, ventricular tachycardia, ventricular fibrillation, and asystole ECG [8-10]. Conduction slowing can often manifest as widening of the QRS representing right bundle or left bundle morphology in the setting of supraventricular tachycardia, which can make recognition difficult and lead to inadequate treatment.

Lipid emulsion therapy was traditionally used in the treatment of anesthetic toxicity based on the hypothesis that lipid emulsion works as a “lipid sink” in which it pulls lipophilic substances from receptor sites in hopes of alleviating the tissues where the toxins mediate their effects. It has also been postulated that lipid emulsion works by increasing voltage-gated calcium channels, which increases the inotropic effect of cardiac myocytes and subsequent faster fatty acid metabolism [5,9]. At the time of writing the current article, there are no randomized controlled trials testing the efficacy of lipid emulsion for use of flecainide toxicity. To our knowledge, our case is the seventh case reported in the literature to document the use of lipid emulsion in the treatment of flecainide toxicity.

The current standard is to use 20% lipid emulsion with 1.5 ml/kg as an initial bolus followed by an infusion of 15 ml/kg/hour [5]. Our patient received sodium bicarbonate infusion on day one but remained bradycardic in the 40s. On day two, he was continued on the bicarbonate drip and was also started on standard lipid emulsion therapy after cardiac arrest (similar to the six prior documented cases) secondary to ventricular tachycardia but remained bradycardic. On day three of admission, the ECG showed atrial fibrillation with slow ventricular response in the 50s, and on day four, he was in sinus rhythm with first-degree AV block with rates in the 70s. 

In cases where lipid emulsion was used [4,6,9,10], it was initiated in the standard dose after a cardiac arrest, as in our case. Extracorporeal life support was used in conjunction with lipid emulsion in the cases by Vu et al. [3], Brumfield et al. [4], Judge [6], Schyver et al. [9], and Sivaligam et al. [10]. The case by Schryver et al. was unique in that two lipid boluses were administered in addition to continuous venovenous hemodiafiltration (CVVHDF).

While lipid emulsion was used in all of these cases after a cardiac arrest, it is impossible to delineate the direct effect it had in all of these cases due to the fact that other treatments (i.e. activated charcoal, magnesium, etc) were used in conjunction. In the case by Smith et al., mere discontinuation of flecainide led to improvement in the ECG and patient status [11]. There are also other cases, as in the case by Ghataoura et al. [5] where sodium bicarbonate was used without lipid emulsion; the ECG normalized and the patient survived.

It is important to note that in the case review done by Valentino et al., deaths were only reported when the patient presented with a QRS > 200 ms [12]. These patients were also more likely to have left bundle branch block (LBBB) on ECG. The use of mechanical circulatory support was also more prevalent in patients with a QRS > 200 ms. It is interesting to note that patients with a QRS < 200 ms were more likely to present with an RBBB on ECG and have a rapid recovery, as in our patient and in the cases by Ghataoura et al. [5] and Venkataraman et al [13]. It is possible that the presence of an RBBB may have been a positive prognostic indicator in these cases; however, in the cases of Sivalingam et al. [10], Schryver et al. [9], Judge [6], Brumfield et al. [4], and Vu et al. [3], these patients survived even though they required extracorporeal life support. Further investigation into the prognostication of QRS duration may prove to be helpful in predicting mortality in flecainide overdose.

## Conclusions

This case of acute flecainide toxicity illustrates several key points. Prompt recognition of clinical and electrocardiographic manifestations of flecainide toxicity is important to establish the correct diagnosis and appropriate management. The initial treatment of toxicity is an infusion of sodium bicarbonate infusion to acutely reverse the effect of flecainide in order to prevent life-threatening arrhythmias. In our case, the patient was not responding to the initial therapy and we decided to treat with lipid emulsion therapy. Currently, there is no recommended standardized treatment protocol for flecainide toxicity. Ultimately, well-designed clinical trials will be required to delineate the true effect of lipid emulsion, used alone or in conjunction with other treatments, to determine its true efficacy in treating flecainide toxicity.
